# Anti-oxidative effects of the biennial flower of *Panax notoginseng *against H_2_O_2_-induced cytotoxicity in cultured PC12 cells

**DOI:** 10.1186/1749-8546-5-38

**Published:** 2010-10-28

**Authors:** Roy Chi-Yan Choi, Zhiyong Jiang, Heidi Qun Xie, Anna Wing-Han Cheung, David Tai-Wai Lau, Qiang Fu, Tina Tingxia Dong, Jijun Chen, Zhengtao Wang, Karl Wah-Keung Tsim

**Affiliations:** 1 Center for Chinese Medicine and Department of Biology, Hong Kong University of Science and Technology, Clear Water Bay, Kowloon, Hong Kong SAR, China; 2 State Key Laboratory of Phytochemistry and Plant Resources in West China, Kunming Institute of Botany, Chinese Academy of Sciences, Kunming, Yunnan 650204, China; 3 Institute of Chinese Materia Medica, Shanghai University of Traditional Chinese Medicine, 1200 Cailun Road, Zhangjiang Hi-Tech Park, Shanghai 201203, China

## Abstract

**Background:**

*Radix notoginseng *is used in Chinese medicine to improve blood circulation and clotting; however, the pharmacological activities of other parts of *Panax notoginseng *have yet to be explored. The present study reports the anti-oxidative effects of various parts of *Panax notoginseng*.

**Methods:**

Various parts of *Panax notoginseng*, including the biennial flower, stem-leaf, root-rhizome, fiber root and sideslip, were used to prepare extracts and analyzed for their anti-oxidation effects, namely suppressing xanthine oxidase activity, H_2_O_2_-induced cytotoxicity and H_2_O_2_-induced ROS formation.

**Results:**

Among various parts of the herb (biennial flower, stem-leaf, root-rhizome, fiber root and sideslip), the water extract of the biennial flower showed the strongest effects in (i) inhibiting the enzymatic activity of xanthine oxidase and (ii) protecting neuronal PC12 cells against H_2_O_2_-induced cytotoxicity. Only the water extracts demonstrated such anti-oxidative effects while the ethanol extracts did not exert significant effects in suppressing xanthine oxidase and H_2_O_2_-induced neuronal cytotoxicity.

**Conclusions:**

The present study demonstrates the biennial flower of *Panax notoginseng *to have neuroprotection effect on cultured neurons and the underlying protection mechanism may involve anti-oxidation.

## Background

*Radix Notoginseng *(*Sanqi*, the root of *Panax notoginseng*) is a Chinese herbal medicine used in China to promote blood circulation, remove blood stasis, induce blood clotting, relieve swelling and alleviate pain [1,2]. Moreover, *Panax notoginseng *is beneficial for coronary heart disease, cerebral vascular disease as well as learning and memory improvement [3-7]. These therapeutic effects are attributed to its active ingredients, namely saponins [8,9], flavonoids [10] and polysaccharides [11,12].

Saponins isolated from *Radix Notoginseng *increase the blood flow of coronary arteries [13], prevent platelet aggregation [14], decrease oxygen consumption by heart muscles [15], restore learning impairment induced by chronic morphine administration [16] and protect neuronal cell death against oxidative stress [17]. Flavonoids increase the coronary flow, reduce myocardial oxygen consumption and lower arterial pressure [10]. A flavonol glycoside called quercetin 3-O-β-D-xylopyranosyl-β-D-galactopyranoside (RNFG) from the root and rhizome of *Panax notoginseng *is promising in treating Alzheimer's disease through inhibiting amyloid-β aggregation and amyloid-β-induced cytotoxicity in cortical neuron cultures. Such neuroprotection effect was mediated by the suppression of apoptosis triggered by amyloid-β [18]. Moreover, polysaccharide extracted from the root-rhizome of *Panax notoginseng *is also considered to be an active constituent with immuno-stimulating activities *in vitro *[11,12,19].

While the therapeutic effects of the root of *Panax notoginseng *have been demonstrated, the pharmacological effects of other parts of *Panax notoginseng *are largely unknown. The present study examines the anti-oxidation effects of other parts of *Panax notoginseng*.

## Methods

### Plant materials and preparation

Fresh *Panax notoginseng *from Wenshan in Yunnan Province (China) was identified morphologically during harvest. Voucher specimen (number 03-6-8) of *Panax Notoginseng *was confirmed by genetic analysis [20] and deposited at the Department of Biology, Hong Kong University of Science and Technology. For water extraction, the biennial flower, stem and leaf, root-rhizome, fiber root and/or sideslip (10 g) were boiled in 80 ml of water for two hours twice. The extract was then dried by lyophilization with an extraction efficiency of 15-18%. For ethanol extraction, biennial flower (10 g) was sonicated in 100 ml of 30%, 50%, 70% and 90% ethanol for 30 minutes twice. The extract was dried by rota-evaporation at 60°C with an extraction efficiency of 5-8%. The water and ethanol extracts were re-dissolved in water to 100 mg/ml stock concentration.

### Cell culture

Rat pheochromatocytoma PC12 cell line was obtained from ATCC (CRL-1721; USA). The cells were maintained in Dulbecco's modified Eagles medium (DMEM) supplemented with 6% fetal bovine serum and 6% horse serum at 37°C in a water-saturated 7.5% CO_2 _incubator. Reagents for cell cultures were purchased from Invitrogen Technologies (USA).

### In vitro xanthine oxidase activity

Xanthine oxidase activity assay was described previously [21]. In brief, the herbal extracts (0.1 mg/ml) were pre-mixed with 0.05U/ml xanthine oxidase for 20 minutes. Then 0.4 mM xanthine and 0.24 mM hydroxyl amine were incubated for 20 minutes at 37°C. Reactions were stopped by adding 0.1% SDS to the mixture and measured at 550 nm absorbance. Vitamin C at various concentrations (0, 17.6, 35.2, 52.8 and 88 μg/ml) served as the positive control of anti-oxidation. All the chemicals were purchased from Sigma (USA).

### Cell viability test

Cultured PC12 cells in 96-well-plate (5000 cells/well) were pre-treated with various extracts (1 mg/ml) for 24 hours. After washed with PBS and replaced by fresh culture medium, the cultures were treated with 13.6 μg/ml hydrogen peroxide (H_2_O_2_) for 24 hours. Cell viability test was performed with the addition of thiazolyl blue tetrazolium bromide (MTT) (Sigma, USA) in PBS at a final concentration of 5 mg/ml for four hours. After the solution was removed, the purple precipitate inside the cells was re-suspended in DMSO and then measured at 570 nm absorbance [22]. H_2_O_2 _at various concentrations (0, 1.7, 3.4, 6.8 and 13.6 μg/ml) served as a control for the cytotoxicity test.

### Determination of ROS formation

The reactive oxygen species (ROS) level in cell cultures was determined according to the method by Zhu *et al*. [22]. Cultured PC12 cells in 96-well-plate were pre-treated with the water and ethanol extracts of biennial flower (1 mg/ml) for 24 hours, and then the cells were labeled by 100M dichlorofluorescin diacetate (DCFH-DA, Sigma, USA) in HBSS for one hour at 25°C. Cultures were treated with 13.6 μg/ml H_2_O_2 _for one hour. The amount of intracellular H_2_O_2_-induced ROS was detected by fluorometric measurement with excitation at 485 nm and emission at 530 nm (SPECTRA max^® ^GEMINI XS, Molecular Devices Corporation, USA).

### Statistical analysis

Individual data were expressed as mean ± standard deviation (SD). A *post-hoc *Dunnett's test was used to obtain corrected P values in group comparisons. Statistical analyses were performed with one-way ANOVA (version 13.0, SPSS, USA). Data were considered as significant when *P *< 0.05 and highly significant when *P *< 0.001.

## Results

### Anti-oxidative effects of Panax notoginseng's biennial flower

**Figure 1:**
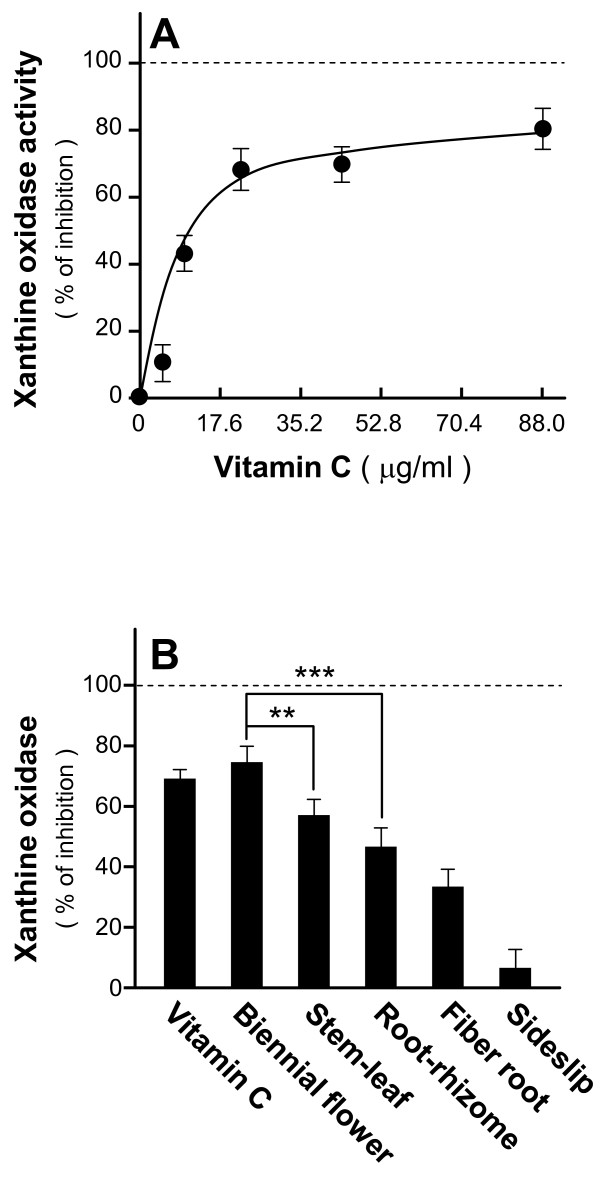
***In vitro *anti-oxidative effects of extracts from various parts of *Panax notoginseng***. **A**: Vitamin C at various concentrations (0, 17.6, 35.2, 52.8 and 88 μg/ml) was pre-incubated with xanthine oxidase before the addition of the xanthine substrate. The xanthine oxidase activity was measured at 550 nm absorbance. **B**: Extracts (0.1 mg/ml) from the biennial flower, stem-leaf, rhizome and fiber root of *Panax notoginseng *were assayed for their anti-xanthine oxidase activity as in [A]. Vitamin C (35.2 μg/ml) served as positive control. Data were expressed as% of inhibition where all the values were normalized by the control (no drug treatment), Mean ± SD, *n *= 6. Statistical significance is indicated as ** *P *= 0.00876 for biennial flower vs stem-leaf; and *** *P *= 0.000586 for biennial flower vs root-rhizome.

**Figure 2:**
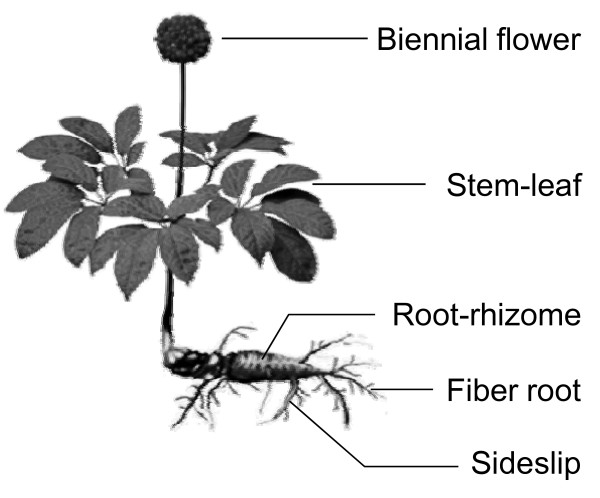
**A schematic diagram to illustrate various parts of *Panax notoginseng***.

To reveal the anti-oxidative effects of *Panax notoginseng*, we carried out an *in vitro *assay of xanthine oxidase effects. The abnormality of the xanthine oxidase causes pathological disorders [23-25]; thus, the enzyme is a biological marker for anti-oxidative effects. In the presence of vitamin C at various concentrations (0, 17.6, 35.2, 52.8 and 88 μg/ml), xanthine oxidase effects were suppressed in a dose-dependent manner, with maximum inhibition of 80% as compared with the control (Figure 1A), validating this anti-oxidation assay. Different parts of *Panax notoginseng *including the biennial flower, stem-leaf, root-rhizome, fiber root and sideslip were separated from the whole plant (Figure 2) and subjected to water extraction. Individual extract was tested on its anti-oxidation effects against xanthine oxidase. Water extract (0.1 mg/ml) from the biennial flower possessed the strongest anti-oxidative effects (about 80% of enzyme inhibition) among various parts of *Panax notoginseng *while the extract from sideslip showed the least effects (Figure 1B). Vitamin C (35.2 μg/ml) served as a positive control with an inhibition rate of about 70%. These results suggested that different parts of *Panax notoginseng *all possessed anti-oxidative effects with varying degrees.

**Figure 3:**
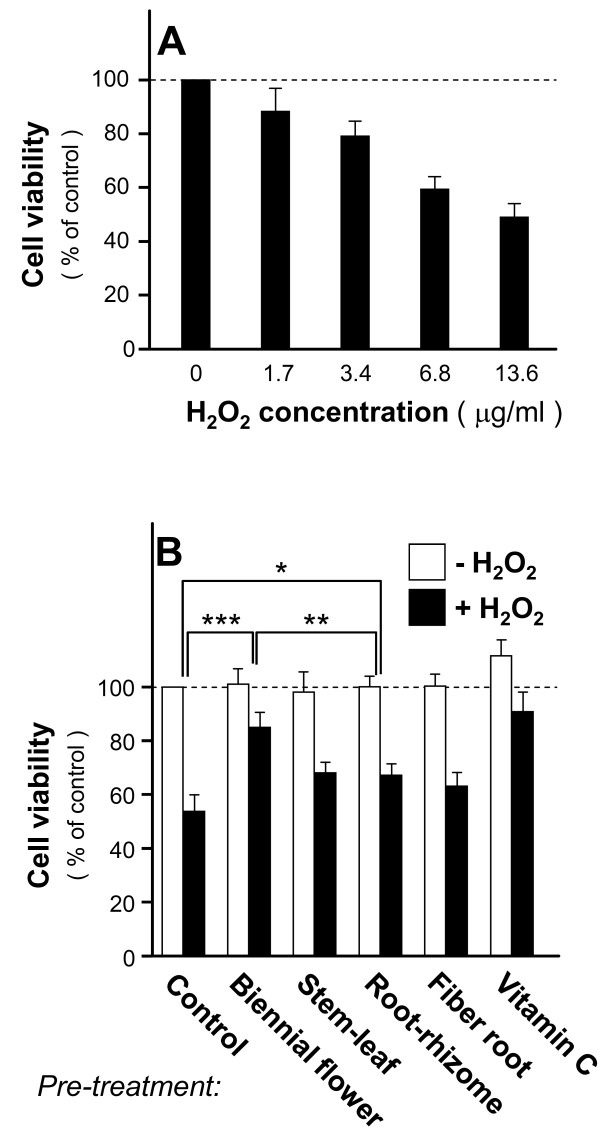
**Anti-oxidative effects by the extract of the biennial flower of *Panax notoginseng *against H_2_O_2_-induced cytotoxicity in PC12 cells**. **A**: Various concentrations of H_2_O_2 _(0, 1.7, 3.4, 6.8 and 13.6 μg/ml) were added onto cultured PC12 cells, incubated for 24 hours and determined with cell viability assay. **B**: Extracts (1 mg/ml) from biennial flower, stem-leaf, rhizome and fiber root of *Panax notoginseng *were pre-treated with PC12 cells for 24 hours before the addition of H_2_O_2 _(13.6 μg/ml) for cytotoxicity test as in [A]. Vitamin C (35.2 μg/ml) served as a positive control. Data were expressed as% of control where the value of untreated culture was set as 100%, Mean ± SD, *n *= 4. Statistical significance is indicated as * *P *= 0.0412 for root-rhizome vs control); ** *P *= 0.00826 for biennial flower vs root-rhizome and *** *P *= 0.000215 for biennial flower vs control.

The above *in vitro *anti-oxidative effects of *Panax notoginseng *could be mediated by a direct interaction between the herb-derived active ingredient(s) and xanthine oxidase. However, we speculate that such interaction may not be allowed inside the cell because the cell permeability and cellular absorption of the active ingredients are unknown. For this reason, a cell-based assay using neuronal PC12 cell was employed. PC12 cell is a popular study model in analyzing the neuroprotective effects against oxidation and other insults [22,26,27]. To inducing oxidative stress, we treated the cultures with various concentrations of H_2_O_2 _(0-13.6 μg/ml) and assayed for their cell viability. The neuronal cytotoxicity of PC12 cells induced by H_2_O_2 _was demonstrated by a dose-dependent decrease of cell viability (Figure 3A). At 13.6 μg/ml concentration of H_2_O_2_, about 50% cells survived. Under such cytotoxic condition, pre-treatment of the extracts from the biennial flower, stem-leaf and rhizome (1 mg/ml) protected PC12 cells against H_2_O_2 _insult (Figure 3B). Among all the tested extracts, the neuroprotective effects of the biennial flower were more robust than those of stem-leaf and rhizome. On the other hand, the extract of fiber root did not show any significant response while the sideslip was not included due to its negative effects in anti-oxidation. Pre-treatment of vitamin C was performed in control. These results showed that the water extract of the biennial flower of *Panax notoginseng *exhibited significant anti-oxidative effects.

### Comparison of anti-oxidative effects by water and ethanol extracts

**Figure 4:**
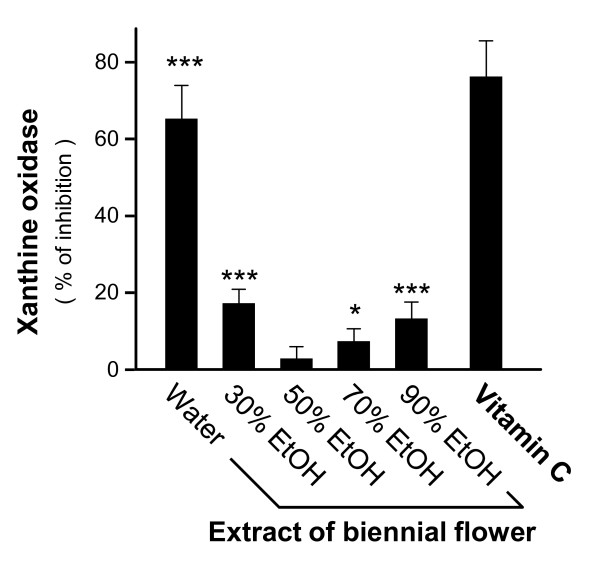
**Comparison of anti-xanthine oxidase effects between the water- and ethanol-extracts of the biennial flower**. Biennial flower of *Panax notoginseng *was extracted by water or various concentrations of ethanol (30, 50 70 and 90%). Extracts (0.1 mg/ml) were tested for their anti-oxidative effects against xanthine oxidase as in Figure 1. Vitamin C (35.2 μg/ml) served as positive control. Data were expressed as% of inhibition where all the values were normalized by the control (no drug treatment), Mean ± SD, *n *= 6. Statistical significance is indicated as * *P *= 0.0419 for control (without extract) vs 70% EtOH and *** *P *= 0.0000852 for control (without extract) vs water, *P *= 0.000725 for control (without extract) vs 30% EtOH and *P *= 0.000897 for control (without extract) vs 90% EtOH.

**Figure 5:**
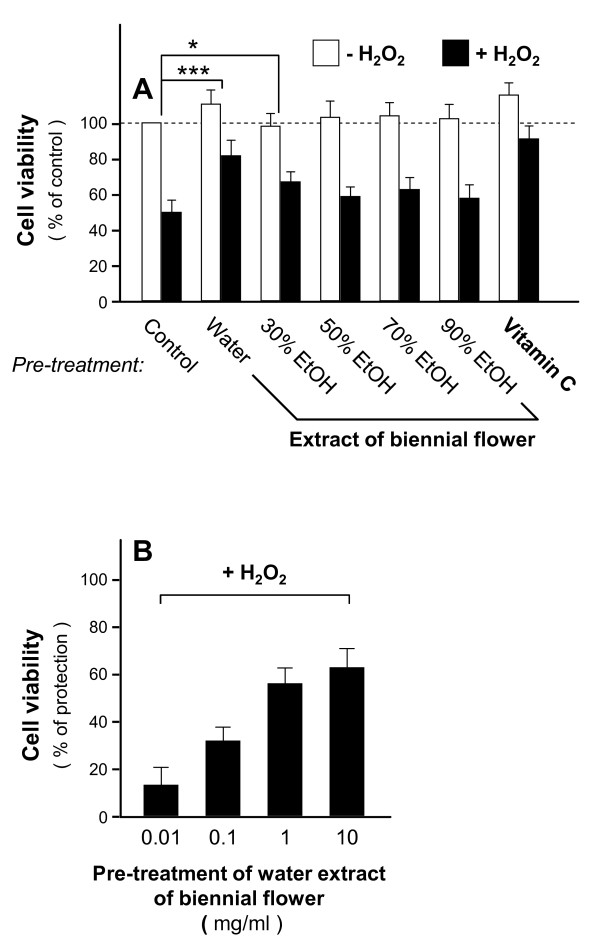
**Dose-dependent effects of the water extract of the biennial flower against H_2_O_2_-induced cytotoxicity in PC12 cells**. **A**: Extracts (1 mg/ml) of biennial flower by water and ethanol extractions were pre-treated with PC12 cells for 24 hours before the addition of H_2_O_2 _(13.6 μg/ml) for cytotoxicity test as in Figure 2. Vitamin C (35.2 μg/ml) served as positive control. **B**: Dose-dependent response was performed by pre-treating the culture with various concentrations of the water extract of the biennial flower (0.01-10 mg/ml). Data were expressed as% of control where the value of untreated culture was set as 100%, Mean ± SD, *n *= 4. Statistical significance is indicated as * *P *= 0.00471 for control (without extract) vs 30% EtOH and *** *P *= 0.000693 for control (without extract) vs water.

To reveal the importance of solvent selection, we used various concentrations of ethanol (30%, 50%, 70% and 90%) in the extraction of the biennial flower. The anti-oxidative effects of the ethanol extracts (0.1 mg/ml) were compared with those of water extraction.The ethanol extracts of the biennial flower showed lesser anti-oxidative effects (Figure 4); both 30% and 90% ethanol extracts exerted about 18% inhibition whereas 50% ethanol extract did not show inhibition at all. Vitamin C served as positive control. Moreover, the neuroprotective effects of the ethanol extracts were tested in cultured PC12 cells. Pre-treatments of 50%, 70% and 90% ethanol extracts did not protect the neuronal cultures against H_2_O_2_-induced cell death (Figure 5A) while 30% ethanol extract slightly exerted neuroprotective effects. The water extract performed the best. To further confirm the anti-oxidative effects of the water extract in PC12 cells, we pre-treated the cultures with various water extracts (0.01-10 mg/ml) and then with H_2_O_2 _and performed cell viability assay. The survival rate of PC12 cells under H_2_O_2 _insult was improved in a dose-dependent manner (Figure 5B). The saturation dose was at about 1 mg/ml. Therefore, water extracts of the biennial flower showed stronger anti-oxidative effects than ethanol extracts.

**Figure 6:**
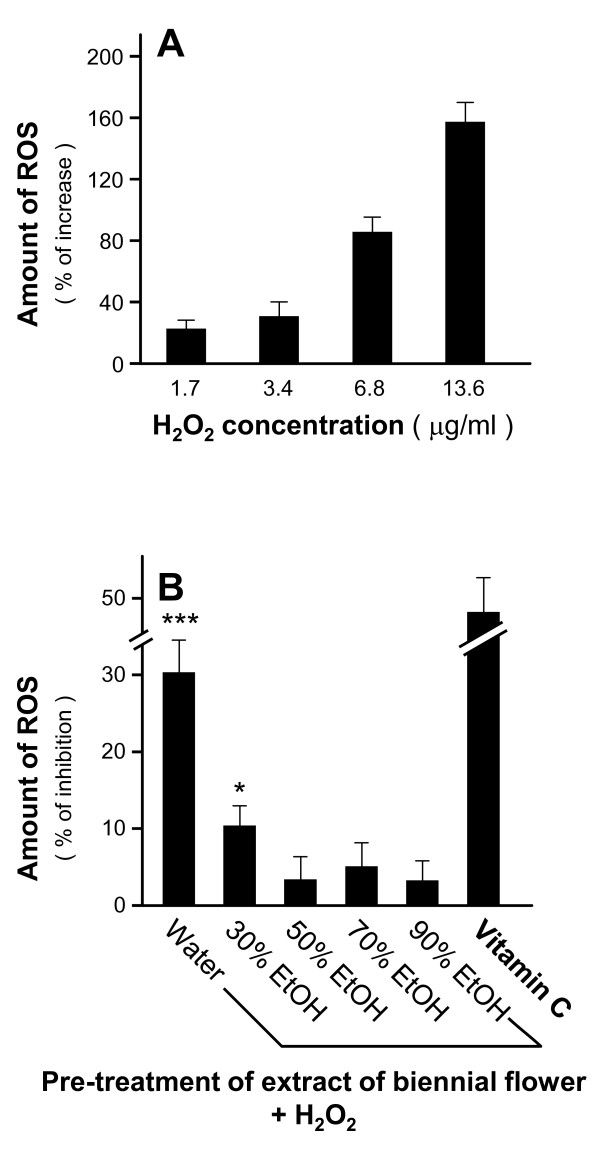
**Suppression of the formation of H_2_O_2_-induced ROS formation in PC12 cells by water extracts of the biennial flower**. **A**: Cultured PC12 cells were pre-labeled DCFH-DA for one hour before the addition of various concentrations of H_2_O_2 _(0, 1.7, 3.4, 6.8 and 13.6 μg/ml) for another hour. The amount of ROS was fluorometrically measured with excitation at 485 nm and emission at 530 nm. **B**: Water and ethanol extracts of the biennial flower (1 mg/ml) were pre-treated with the PC12 cells for 24 hours. H_2_O_2 _(13.6 μg/ml) was used in the ROS formation assay as in A. Vitamin C (35.2 μg/ml) served as positive control. Data were expressed as% of inhibition where all the values were normalized by the control (no drug treatment), Mean ± SD, *n *= 4. Statistical significance is indicated as * *P *= 0.00419 for control (without extract) vs 30% EtOH and *** *P *= 0.000269 for control (without extract) vs water.

To elucidate the anti-oxidative mechanism of the biennial flower, we chose reactive oxygen species (ROS) for the investigation because ROS promote the oxidation of lipid, protein and DNA, thereby affecting the normal cell physiology, leading to neuronal demise [28,29]. Cultured PC12 cells were pre-labeled with an ROS indicator and then treated with various concentrations of H_2_O_2 _(0-400 μM). Upon the addition of H_2_O_2_, ROS formation increased in a dose-dependent manner (Figure 6A). Such elevation of ROS in cultured PC12 cells was reduced by the pre-treatment of water extract of the biennial flower, with about 30% ROS inhibition (Figure 6B). By contrast, 30% ethanol extract slightly reduced the amount of H_2_O_2_-induced ROS whereas 50%, 70% and 90% of ethanol extracts did not show any effects.

## Discussion

The present study, for the first time, demonstrated the anti-oxidative effects possessed by the water extract of the biennial flower of *Panax notoginseng *through the suppression of H_2_O_2_-induced ROS formation and neuroprotection against H_2_O_2 _insult. More importantly, it was the biennial flower instead of the root-rhizome that showed the strongest effects. These results support the multi-functional roles of *Panax notoginseng *and warrant further studies to explore other pharmacological effects of the plant. In terms of identifying the possible active ingredient(s) from the biennial flower, the anti-oxidation effects of different ethanol extracts were shown to be significantly less potent than that of the water extract, suggesting that the majority of active compounds might be preferentially water soluble. However, a continuous work of activity-guided fractionation is required to purify and identify the candidates from the water extract of biennial flower. In this case, the high solubility of those active compounds in water will facilitate the preparation of health food supplements and drinks that could be developed from the biennial flower. Indeed, this new application will increase the economic value of *Panax notoginseng*.

Neuronal action of *Panax notoginseng *on the brain possesses various aspects. Saponins derived from the herb have been shown to prevent the neuronal cell death against hypoxia condition. The mechanism was related to the improvement of energy metabolism [30]. The therapeutic effect of saponins derived from *Panax notoginseng *was further supported by promoting the absorption of hematoma in hemorrhagic apoplexy at super-early stage in rat [31], and protecting the neuron against insults and promoting functional rehabilitation in patients after cerebral hemorrhage [32]. In addition, the co-treatment of icariin and sponins derived from *Panax notoginseng *exerted significant prophylactic and therapeutic effects in rat models of Alzheimer's disease *in vivo *[33], as well as ameliorated the learning and memory deficit and blood viscosity by protecting neurons from oxidative stress in ischemic brain [34]. For neurotrophic effects, the phosphorylated neurofilament- and MAP2-expressing neurites could be extended in SK-N-SH cells by the treatment of saponins and *Panax notoginseng *extracts, suggesting the possible axonal and dendritic formation activity [35]. Therefore, the multi-functional effects of saponins from *Panax notoginseng *might be a good candidate in mediating the anti-oxidation activities because of the high extractability of saponins by water. This speculation was in accordance to our previous finding that the amounts of four active constituents, notoginsenoside R_1_, ginsenoside R_g1_, R_b1 _and R_d_, by water extraction were higher than that of 30% and 70% ethanol extractions [36]. In addition to saponins, a flavonol glycoside, named RNFG, isolated from *Panax notoginseng *also possesses the neuroprotective effect against amyloid-β-induced apoptosis and cytotoxicity at cellular level, and which improves the learning and memory process in rats [18]. Interestingly, this compound also exerts a significant anti-oxidative activity by lowering the amount of reactive oxygen species (ROS) induced by H_2_O_2 _in cultured PC12 cells. Based on the above findings, it should be very interesting to know if the biennial flower contains RNFG, and which could have neuroprotective effect in cell cultures and in animal study. Therefore, the identification and isolation of the possible active ingredients (saponins, flavonoids, flavonol glycoside or others) will be essential to extend and support the multi-functional usages of *Radix Notoginseng *in future.

## Conclusion

The present study demonstrates the biennial flower of *Panax notoginseng *to have neuroprotection effect on cultured neurons and the underlying protection mechanism may involve anti-oxidation.

## Abbreviations

CO_2_: carbon dioxide; DCFH-DA: dichlorofluorescin diacetate; DMEM: Dulbecco's modified eagle medium; DMSO: dimethyl sulfoxide; H_2_O_2_: hydrogen peroxide; HPLC: high performance liquid chromatography; MTT: 3-(4,5)-dimethylthiahiazo (-z-y1)-3,5-di- phenytetrazoliumromide; PBS: phosphor-buffer saline; RNFG: 3-O-β-D-xylopyranosyl-β-D-galactopyranoside; ROS: reactive oxygen species; SDS: sodium dodecyl sulfate

## Competing interests

The authors declare that they have no competing interests.

## Authors' contributions

TTXD, JJC, ZTW and KWKT designed the experiments. RCYC, ZYJ, HQX, AWHC, DTWL and QF conducted the experiments. All authors read and approved the final version of the manuscript.
